# Plant and algal lysophosphatidic acid acyltransferases increase docosahexaenoic acid accumulation at the *sn-*2 position of triacylglycerol in transgenic Arabidopsis seed oil

**DOI:** 10.1371/journal.pone.0256625

**Published:** 2021-08-25

**Authors:** Laura L. Wayne, Daniel J. Gachotte, Paul R. Graupner, Yelena Adelfinskaya, David G. McCaskill, James G. Metz, Ross Zirkle, Terence A. Walsh

**Affiliations:** 1 Corteva Agriscience, Johnston, Iowa, United States of America; 2 Corteva Agriscience, Indianapolis, Indiana, United States of America; 3 DSM Nutritional Products, Columbia, Maryland, United States of America; Texas A&M University College Station, UNITED STATES

## Abstract

Although docosahexaenoic acid (DHA), an important dietary omega-3 polyunsaturated fatty acid (PUFA), is at present primarily sourced from marine fish, bioengineered crops producing DHA may offer a more sustainable and cost-effective source. DHA has been produced in transgenic oilseed crops, however, DHA in seed oil primarily occupies the *sn-*1/3 positions of triacylglycerol (TAG) with relatively low amounts of DHA in the *sn-*2 position. To increase the amount of DHA in the *sn-*2 position of TAG and in seed oil, putative lysophosphatidic acid acyltransferases (LPAATs) were identified and characterized from the DHA-producing alga *Schizochytrium* sp. and from soybean (*Glycine max*). The affinity-purified proteins were confirmed to have LPAAT activity. Expression of the *Schizochytrium* or soybean LPAATs in DHA-producing Arabidopsis expressing the *Schizochytrium* PUFA synthase system significantly increased the total amount of DHA in seed oil. A novel sensitive band-selective heteronuclear single quantum coherence (HSQC) NMR method was developed to quantify DHA at the *sn-*2 position of glycerolipids. More than two-fold increases in *sn-2* DHA were observed for Arabidopsis lines expressing *Schizochytrium* or soybean LPAATs, with one *Schizochytrium* LPAAT driving DHA accumulation in the *sn*-2 position to 61% of the total DHA. Furthermore, expression of a soybean LPAAT led to a redistribution of DHA-containing TAG species, with two new TAG species identified. Our results demonstrate that transgenic expression of *Schizochytrium* or soybean LPAATs can increase the proportion of DHA at the *sn*-2 position of TAG and the total amount of DHA in the seed oil of a DHA-accumulating oilseed plant. Additionally, the band-selective HSQC NMR method that we developed provides a sensitive and robust method for determining the regiochemistry of DHA in glycerolipids. These findings will benefit the advancement of sustainable sources of DHA via transgenic crops such as canola and soybean.

## Introduction

Docosahexaenoic acid (DHA) is an important dietary omega-3 long-chain polyunsaturated fatty acid (LC-PUFA) for human health. Omega-3 LC-PUFAs are associated with reduced risk of cardiovascular disease and DHA is also important for brain and eye development [1, 2]. Consequently, there is increasing demand for DHA that is typically sourced from marine fish. Fish accumulate DHA via the food chain from marine algae primary producers. These algae can synthesize DHA either by an oxygen-dependent fatty acid desaturation and elongation pathway that is widespread in nature or via a unique anaerobic polyunsaturated fatty acid (PUFA) synthase system that has similarities to microbial polyketide synthases [3]. A *Schizochytrium* sp. used for commercial DHA production uses this PUFA synthase system to produce DHA and other omega-3 LC-PUFAs [4], and accumulates 35% of the total oil fatty acids as DHA [5, 6]. Marine algal fermentation is relatively costly, so this DHA oil is primarily marketed into high-value supplement markets. Conventional omega-3 LC-PUFA sourcing from farmed and wild fish stocks has increasing production and sustainability limitations [7], and the potential for contamination by accumulated heavy metals in fish. Therefore, alternative sustainable and cost-effective sources of DHA by producing omega-3 LC-PUFAs in oilseed crops are of great commercial interest [8].

Production of DHA is successfully exemplified in the model oilseed plant Arabidopsis [9, 10] and translated into oilseed crops *Camelina sativa* [10–12] and canola [13]. This biotechnological advancement was achieved by expression of a suite of elongases and oxygen-dependent desaturases comprising six or more transgenes from fungal and algal sources that provides a pathway from native linoleic acid through to DHA. The alternative anaerobic PUFA synthase route to DHA has also been tested in Arabidopsis and introduced into the major oilseed crops canola and soybean to produce DHA at somewhat lower levels than the elongase/desaturase pathway, but with the advantage of complementing the fatty acid profile and accumulating fewer intermediary LC-PUFAs [14, 15]. Algal PUFA synthase systems consist of three proteins required for the synthesis of DHA directly from malonyl-CoA; PFA1, PFA2, and PFA3 [4]. A phosphopantetheinyl transferase, which activates the acyl carrier protein modules in PFA1 [6], is also required for functionality. Expression of these four proteins in canola and soybean seeds leads to accumulation of up to 4% DHA in seed oil [14].

Direct *de novo* triacylglycerol (TAG) synthesis in plants involves three sequential acylations of glycerol via three acyltransferases known as the Kennedy pathway. In this pathway, lysophosphatidic acid acyltransferase (LPAAT; EC 2.3.1.51) acylates lysophosphatidic acid at the *sn-*2 position with an acyl-CoA to yield phosphatidic acid (PA). TAG can also be synthesized through non-linear pathways involving the phosphatidylcholine (PC) pool in the endoplasmic reticulum (ER) membrane. The various routes and fluxes through these pathways to TAG are species dependent (see review [16]). Transgenic expression of native or heterologous acyltransferases can increase total oil content [17], modify fatty acid composition [18], or increase production of novel non-native fatty acids [19, 20] in seed oils. Here, we focus on limitations associated with the transfer of DHA into the *sn-*2 position of TAG by LPAAT in transgenic DHA-accumulating Arabidopsis plants.

Transgenic expression of plant and fungal LPAATs can increase the accumulation of desired fatty acids at the *sn-*2 position of TAG in oilseeds [20–24]. The acyl-CoA substrate preferences of the specific LPAATs chosen for expression in transgenic plants are key in directing the desired novel fatty acids into TAG. A putative LPAAT from *Thraustochytrium* sp. was tested in DHA-accumulating transgenic *B*. *juncea*, although its contribution to DHA production is negligible [25]. Some fungal LPAATs have selectivity for DHA-CoA, as expression of *Mortierella alpina* LPAAT in Arabidopsis seeds increases the proportion of DHA content at the *sn-2* position of TAG and the total DHA content in transgenic DHA-accumulating Arabidopsis with the elongase-desaturase DHA pathway [26]. However, to our knowledge, no plant or algal LPAATs with DHA specificity have been found to increase DHA incorporation at the *sn*-2 position of glycerolipids in transgenic oilseeds.

A challenge in these types of studies is the ability to determine the regiochemistry of DHA-containing TAG species in seed oils. Current methods include digestion of glycerolipids via position-specific lipases followed by fatty acid analysis or use of nuclear magnetic resonance (NMR) spectroscopic analyses of seed oil. Carbon-13 NMR methods detect minimal DHA at the *sn-*2 position of TAG in DHA-accumulating transgenic Arabidopsis and *Camelina* [9, 11]. The lipase digestion method was used to determine that transgenic LC-PUFA-accumulating *Camelina* has three-fold enrichment of DHA and eicosapentaenoic acid (EPA) at the *sn-*1/*sn-*3 positions over the *sn-*2 position of TAG [10]. Transgenic LC-PUFA-accumulating *B*. *juncea* has predominately *gamma*-linolenic acid and arachidonic acid at the *sn-*2 position of TAG determined by lipase digestion [25]. Expression of the *M*. *alpina* LPAAT increases *sn*-2 DHA in seed oil, which was confirmed by both lipase digestion and ^13^C NMR [26]. Nevertheless, a more sensitive and convenient analytical method to quickly screen for and quantify *sn*-2 DHA will greatly aid characterization of DHA-containing glycerolipids and functional analysis of LPAATs that can increase *sn*-2 DHA in seed oils.

In this study, we have identified LPAATs from soybean and *Schizochytrium* that increased the total amount of DHA in seed oil from transgenic DHA-accumulating Arabidopsis and substantially increased the proportion of DHA in the *sn-*2 position of TAG. These enzymes represent the first characterized plant and algal LPAATs to our knowledge that enhance the accumulation of *sn-*2 DHA in transgenic plants. We also describe a two-dimensional (2-D) NMR method to determine the position of DHA in TAG, which enabled transgenic LPAAT lines to be screened and prioritized. SzLPAAT3 increased the relative amount of DHA at the *sn-*2 position to almost two-thirds of the total DHA. Furthermore, two novel DHA-containing TAG species were identified in oil from soybean LPAAT events, demonstrating the *in vivo* selectivity of a heterologous plant LPAAT enzyme to alter the acyl-chain distribution of TAG. Our results indicate that plant and algal LPAATs can enhance *sn-*2 DHA through regiospecific changes to DHA TAG in transgenic Arabidopsis and have the potential to increase total DHA and *sn-*2 DHA in transgenic oilseed crops.

## Materials & methods

### LPAAT protein expression

The LPAAT genes were transfected into Sf9 cells using the BacPAK^™^ Baculovirus expression system (Takara Bio). Titer determination was performed by Expression Systems LLC. To scale up for purification, High Five (H5) cells were seeded into 1L shake cultures at approximately 1.5 x 10^6^ cells/mL and grown overnight at 27°C and 135 rpm. The following day, the cells were counted and BacPAK9/LPAAT virus was added when multiplicity of infection = 1. Incubation was continued at 27°C and 135 rpm for 48 hours. Transfected cells were pelleted and stored at -80°C until further use.

### LPAAT protein purification

His-tagged LPAAT proteins were purified with a cobalt metal affinity chromatography (CoMAC) resin (Takara Bio). Cells were resuspended in lysis buffer (50 mM potassium phosphate pH 8.0, 100 mM KCl, 10% glycerol, and 1 mM TCEP) and sonicated. Lysates were then centrifuged at 18,000 x g for 15 min at 4°C. Microsomes were prepared by centrifuging the supernatant at 100,000 x g for 1 h at 4°C. The microsome pellets were resuspended in ~2 mL of 50 mM potassium phosphate pH 8.0 with 100 mM KCl, 10% glycerol, 1 mM TCEP, and 1% DDM (n-dodecyl β-D-maltoside) detergent and incubated on ice for 30 min. Samples were centrifuged at 18,000 x g for 20 min at 4°C and the supernatant was diluted to 5 mL in buffer without detergent. The CoMAC resin was equilibrated with two volumes of equilibration/wash buffer (50 mM potassium phosphate pH 8.0, 100 mM KCl, 10% glycerol, 1 mM TCEP, 0.05% DDM detergent, and 10 mM imidazole) and the diluted supernatant microsomes were applied to the resin and rocked for 1.5 hours at 4°C. The column was then washed with two volumes of wash buffer. The His-tagged LPAAT proteins were eluted with four volumes of elution buffer (50 mM potassium phosphate pH 8.0, 100 mM KCl, 10% glycerol, 1 mM TCEP, 0.05% DDM detergent, 250 mM imidazole). Protein concentrations were measured using a Bradford assay or Nanodrop spectrophotometer (Thermo Fisher) and visualized via SDS-PAGE and western blot using a monoclonal anti-His antibody conjugated to alkaline phosphatase. Aliquots were flash frozen in liquid nitrogen and stored at -80°C.

### LPAAT *in vitro* assays

The *in vitro* enzyme assay reaction consisted of 50 mM Tris-HCl pH 7.5, 40 μM lysophospholipid, 1 mM MgCl_2_, 18 μM [^14^C] acyl-CoA (American Radiolabeled Chemicals) and was initiated by addition of 20 μg of microsomal protein or approximately 100 ng of purified protein. The reaction proceeded for 5 minutes at room temperature. The reaction was quenched by the addition of 2 mL of chloroform:methanol (1:1 v/v) followed by 1 mL of 1 M KCl in 0.2 M H_3_PO_4_, similar to methods previously described [27]. The organic bottom phase was transferred to a new tube and dried down under nitrogen. The samples were resuspended in 130 μL of chloroform:methanol:water (73:23:1 v/v) containing 0.01% BHT. The phospholipids were separated by an Agilent HPLC (1100 series) equipped with two coupled 100 x 4.6 mm Onyx monolithic silica (Phenomenex) columns, with 50 μL of the sample injected. Mobile phase A was acetonitrile:tetrahydrofuran (70:30 v/v) and mobile phase B was 100 mM ammonium formate titrated with formic acid to pH 3.4. The flow rate was 2 mL/min with a gradient as follows: 9.5% B, 15% B at 5 min, 25% B at 8.2 min, 9.5% B at 8.3 min, and 9.5% B at 10 min, and a column temperature of 30°C. The radiolabeled products were detected with a flow scintillation analyzer (Perkin Elmer).

### Plant growth

Arabidopsis seeds were cold stratified for 48 hours and were germinated in a growth chamber (25ᵒC, 16h/8h day/night cycle) for 7 days. Seedlings were then either thinned or transplanted to single plants per pot and transferred to the greenhouse, with 18h artificial light at 23°C. Plants transformed via *Agrobacterium*-mediated transformations were not thinned and floral dipping was conducted as described previously [14]. Transformed seeds were selected by 2,4-dichlorophenoxyacetic acid (2,4-D) [28] application at 7 days post-sowing and again 9 days post-sowing with 107 mg/L 2,4-D at an effective rate of 75 g/ha per application.

### Cloning and molecular detection of transgenes

*Schizochytrium LPAAT* genes [29] were codon optimized for plant expression and synthesized by DNA2.0. Genes were cloned into entry vector cassettes containing the *Phaseolus vulgaris* phaseolin promoter and terminator using In-Fusion^®^ cloning (Takara Bio). Cassettes were then subcloned into binary plant transformation vectors containing RFP and AAD12 [28] selection markers using the Gateway^®^ cloning system (Thermo Fisher). DNA was isolated from Arabidopsis leaf tissue using the BioSprint 96 DNA Plant Kit (Qiagen) extraction method. Transgenes *LPAAT*, *AAD12* (for 2,4-D resistance), and the internal reference gene assay *TafII-15* (accession number: At4g31720) were assayed by real-time PCR using the LightCycler^®^ 480II system (Roche). Primer and probe sequences are listed in S1 Table. Amplification was performed in multiplex in a two-step reaction consisting of an extension at 60°C for 40 seconds followed by fluorescence acquisition.

### LPAAT protein MS detection

Protein extraction was based on a previously established method [30]. Briefly, seeds were homogenized in 10% trichloroacetic acid (TCA) with 0.2% β-mercaptoethanol in acetone using a bead mill to extract protein. TCA was removed with multiple washes of cold acetone. Protein was extracted into phenol and subsequently precipitated with ammonium acetate. Dried protein was solubilized in 8 M urea + 2% β-octylglucoside (BOG), then reduced (25 mM DTT) and alkylated (50 mM iodoacetamide). Each sample was digested with an equal volume of Trypsin/Lys-C mix (Promega) in 100 mM triethylammonium bicarbonate (pH 8.0) for 3 h at 37°C, then diluted with 50 mM TEAB pH 8.0 to a final concentration of 0.8 M urea. Samples were digested at 37°C for an additional 16 h. Digests were quenched with formic acid and desalted into 0.1% formic acid using C18 spin columns (Pierce). Digests were analyzed on a QExactive mass spectrometer (Thermo Scientific) with data acquisition at 70k resolution MS1 and top 15 data dependent MS2 acquisition at 17.5k resolution. Chromatography used an Eksigent nano-LC system (AB Sciex) using a trap and elute chromatographic separation with a C18 ChromXP trap and column operated at 300 nL/min with a water/acetonitrile/0.1% formic acid gradient. Peptides were identified using SEQUEST HT in Proteome Discoverer (v. 1.4, Thermo Scientific) against a uniprot plant sequence database which included the target protein sequences. Label-free relative quantification of the target proteins was carried out using a high-resolution accurate mass MS1 extracted ion chromatogram workflow [31].

### Lipid analysis

Seed oil extraction and analysis of fatty acids were performed as previously described [14]. The lipidomic workflow was developed by Sciex using infusion-based introduction of total lipid extracts into a Sciex 5600 QTOF mass spectrometer [32]. The mass spectrometer instrumental parameters including gas flow and applied voltages were optimized prior to the analyses and the results were normalized using class-specific standard mixes (Avanti Polar Lipids). Data analysis was performed using LipidView^™^ software (Sciex, version 1.3 (beta)), which allowed for the batch processing of TOF-MS and MS/MS data for identification of lipid molecules using a lipid fragment database. The data collected were profiled by searching the lipid fragment database for TAG parent-ion masses using the DHA fragment-ion mass. The parameters used for lipid analysis using Sciex MS/MS^ALL^ infusion method including LC conditions and MS set-up are described in S2 Table.

### NMR spectroscopy

Regiospecific DHA TAG standards were custom synthesized by Larodan. NMR spectra were acquired on a 600 MHz Bruker Avance III NMR spectrometer operating at 600.13 MHz equipped with a 5mm inverse RT probe using standard experiments and parameters provided as part of the TopSpin 3.2 operating system. Samples were dissolved in 600 μl CDCl_3_, and spectra acquired at a temperature of 25°C. For the selective experiments, selective pulses were used in the carbon dimension exciting only a 6-ppm band centered at 34 ppm, designed to give signals from the carbons attached to the acyl end of the triacylglyceride, where 16 transients would be acquired with 256 slices. Linear prediction was used to give a final dataset of 1K x 1K. QSINE window functions were used in both dimensions before Fourier transformation.

## Results

### Qualitative detection of *sn-*2 DHA in transgenic oilseeds

Initially, we used ^1^H NMR to detect DHA at the *sn-*2 position of glycerolipids in transgenic oilseeds and in oil from *Schizochytrium*, the source organism of the PUFA synthase PFA genes. The transgenic plants (canola, soybean, and Arabidopsis) contained the three *Schizochytrium* PFA genes and the *Nostoc* phosphopantetheinyl transferase gene driven by seed-specific promoters [14, 15]. Custom standards of *sn*-1/*sn*-3-DHA and *sn*-2 DHA TAGs were synthesized to validate the ^1^H NMR method (S1A Fig). However, imprecision from using only 1-D spectra was large due to second order effects and so was not reliably quantitative. Quantifying the positional DHA groups by ^1^H NMR was hindered by H-2 and H-3 having very similar chemical shifts, which led to broad non-first order multiplets in one-dimensional proton spectra (S1B Fig).

Oil from *Schizochytrium* was used as a positive control for this ^1^H NMR method where *sn*-2 DHA was detected (S1B Fig) and found to contain 52.1% DHA relative to the total amount of fatty acids in TAG. In contrast, no *sn-*2 DHA was detected from a yeast strain expressing the PUFA synthase genes that produced 16.1% DHA in total glycerolipids (S1B Fig). Of the three transgenic plant species producing DHA that were tested, the DHA soybean lines had clearly detectable levels of *sn-*2 DHA. These soybean lines analyzed by ^1^H NMR had total DHA levels of 5.6% (T_3_ line), 2.9% (T_3_ line), and 4.5% (T_2_ line). In contrast, *sn-2* DHA could not be detected in TAG from DHA-accumulating Arabidopsis lines containing 1.4% (T_2_ line) and 2.9% total DHA (T_3_ line), and a T_2_ canola line containing 1.2% total DHA (S1B Fig). These initial results indicated that soybean and *Schizochytrium* contain acyltransferases that can acylate the *sn-*2 position of TAG with DHA and a more quantitative method for detecting DHA at the *sn*-2 position of TAG would be of great utility.

### Identification of putative LPAAT genes

**Fig 1:**
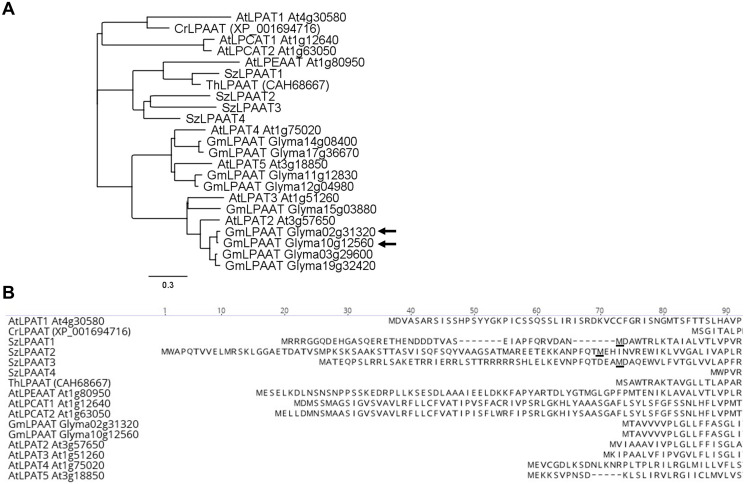
Comparison of LPAAT sequences. **(A)** Phylogenetic analysis of LPAATs using neighbor-joining method from a global alignment with free end gaps created in Geneious v9.1 (Biomatters). Arrows point to the GmLPAAT proteins further characterized in this study. At, *Arabidopsis thaliana*; Cr, *Chlamydomonas reinhardtii*; Gm, *Glycine max*; Sc, *Saccharomyces cerevisiae*; Sz, *Schizochytrium* sp.; Th, *Thraustochytrium* sp. **(B)** Multiple sequence alignment of plant and algal acyltransferase proteins. The underlined methionine residues indicate a potential start site for the SzLPAATs and were used as the initiation codon for the truncated versions.

We focused on identifying LPAATs from *Schizochytrium* and soybean since initial ^1^H NMR results indicated that these two species could produce TAGs containing *sn-2* DHA (S1B Fig). Four putative *LPAAT* genes were identified in the genome of *Schizochytrium* based upon sequence homology to known LPAATs and were named SzLPAAT1, SzLPAAT2, SzLPAAT3, and SzLPAAT4 (Fig 1A; [29]). The putative SzLPAAT proteins were divergent from each other and from other plant LPAATs. The closest protein homolog of SzLPAAT1 was a *Thraustochytrium* LPAAT (75% amino acid identity) but SzLPAAT1 had <30% amino acid identity with the other putative SzLPAATs (Fig 1A). As the initiation codons from the putative SzLPAATs could not be reliably predicted, both full-length (FL) [29] and truncated (tr) versions of SzLPAAT1, SzLPAAT2, and SzLPAAT3 were expressed and analyzed (Fig 1B). The truncated proteins began at Met40 for SzLPAAT1, Met48 for SzLPAAT2, and Met70 for SzLPAAT3 (Fig 1B). As the SzLPAAT4 protein was over 50 amino acids shorter at the N-terminus compared to the other three SzLPAATs, only the FL version was characterized. These seven SzLPAAT sequences were selected for *in planta* characterization. In soybean, nine putative LPAATs were identified by a BLAST search of Soybase [33] using the Arabidopsis LPAT2 sequence [34]. These proteins were aligned with known LPAATs and the putative SzLPAATs (Fig 1). Two of these proteins were expressed higher in the seed during seed filling [35] and were predicted to localize to the ER membrane according to Plant-mPLoc [36]. Therefore, these two putative soybean LPAAT proteins, Glyma**02**g31320 and Glyma**10**g12560, were also chosen for further analysis and referred to as Gm02LPAAT and Gm10LPAAT, respectively.

### Affinity-purified acyltransferases have LPAAT activity

**Fig 2:**
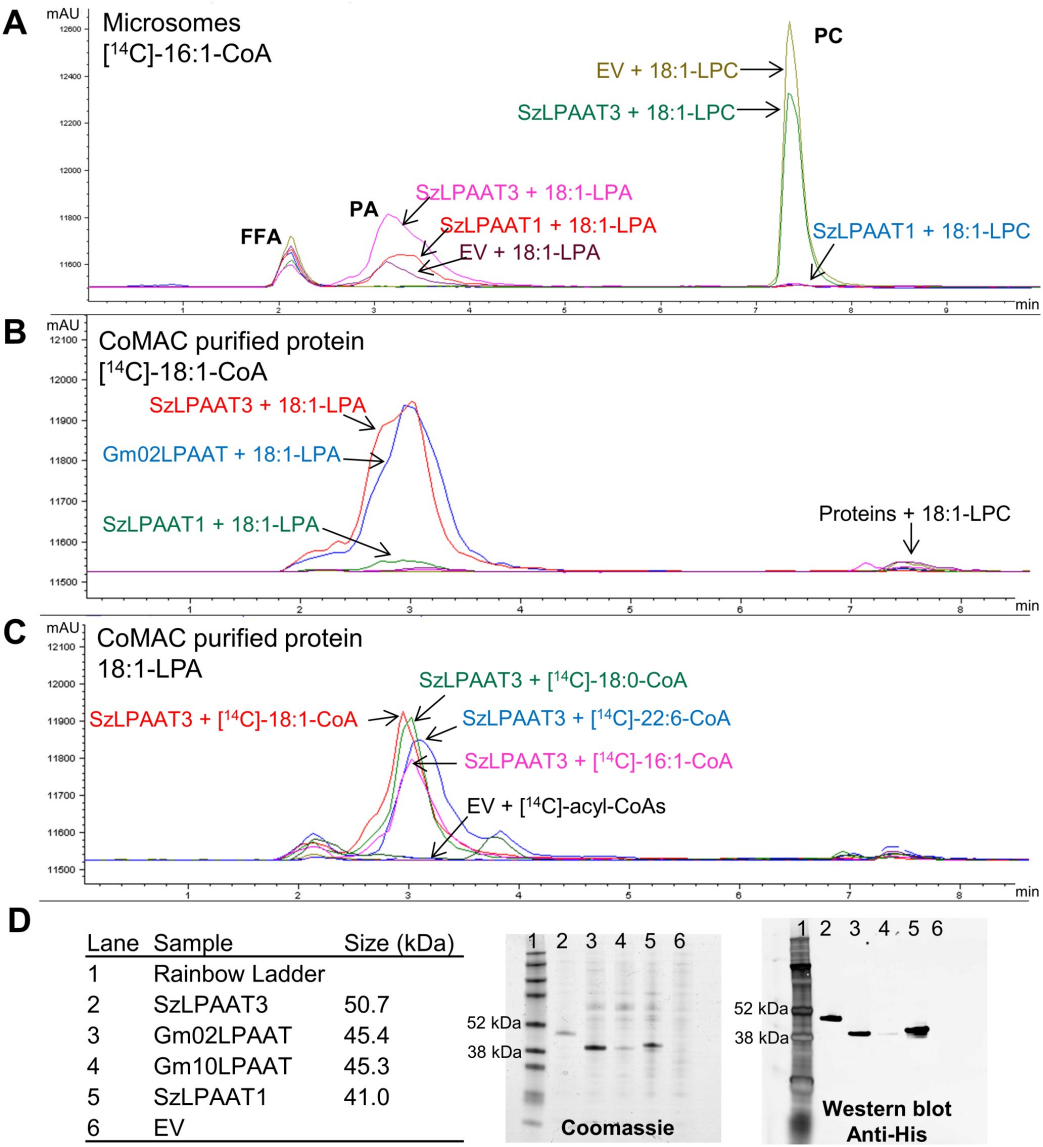
*In vitro* enzyme assays with putative LPAAT proteins expressed in Baculovirus-transfected insect cells. **(A)** Chromatogram overlay of extracts from microsomal prepared fraction assayed with [^14^C]-16:1-CoA using either 18:1-LPA or 18:1-LPC substrate. PC, phosphatidylcholine; PA, phosphatidic acid; FFA, free fatty acid. **(B)** Chromatogram overlay of extracts from CoMAC purified proteins assayed using [^14^C]-18:1-CoA and 18:1-LPA substrates. **(C)** Chromatogram overlay of extracts from CoMAC purified SzLPAAT3 compared with empty vector (EV) controls assayed with four different radiolabeled acyl-CoA substrates. **(D)** Coomassie blue-stained SDS-PAGE gel and western blot of CoMAC purified proteins.

A subset of the putative LPAAT proteins was selected for protein expression and enzymatic characterization; SzLPAAT1 FL, SzLPAAT3 FL, and Gm02LPAAT. These three proteins were expressed using a Baculovirus-insect cell expression system with the High Five (H5) cell line from *Trichoplusia ni*, as this expression system is suitable for expressing lipid-related membrane-bound proteins [37–39]. Microsomal fractions of the SzLPAAT1 and SzLPAAT3 expressed in H5 were assayed for *in vitro* activity. These microsomal preparations had high background activity, especially with oleoyl-lysophosphatidylcholine (18:1-LPC) substrate, which was evident in the empty vector control (Fig 2A). To reduce the amount of background activity (presumably *T*. *ni* endogenous acyltransferase activities) the putative LPAATs, including Gm02LPAAT, were solubilized with n-dodecyl β-D-maltoside (DDM) detergent and purified with cobalt metal affinity chromatography (CoMAC), using the N-terminal His tag on the recombinant proteins. The recombinant proteins from the CoMAC purification were found to have activity with 18:1-LPA and not with 18:1-LPC using [^14^C]-18:1-CoA as substrate, confirming that these three proteins are LPAATs and not lysophosphatidylcholine acyltransferases (LPCATs; Fig 2B). The activity of SzLPAAT1 was low, even though the recombinant protein expressed well in H5 (Fig 2D). SzLPAAT3 was further characterized by assaying with several radiolabeled acyl-CoA substrates, including [^14^C]-18:1-CoA, [^14^C]-18:0-CoA, [^14^C]-16:1-CoA, and [^14^C]-22:6-CoA. SzLPAAT3 had similar relative activities with all four radiolabeled acyl-CoA substrates (Fig 2C), indicating that SzLPAAT3 efficiently incorporates DHA-CoA into phosphatidic acid. These results demonstrate that SzLPAAT1, SzLPAAT3, and Gm02LPAAT are LPAAT enzymes.

### Expression of heterologous LPAATs increases *sn-*2 DHA in Arabidopsis seed TAG

The seven *Schizochytrium* LPAAT sequences and two soybean LPAAT sequences were cloned into plant transformation vectors driven by the strong seed-specific *Phaseolus vulgaris* phaseolin promoter and transformed into Arabidopsis plants expressing the PUFA synthase system via *Agrobacterium*-mediated transformations. The transgenic Arabidopsis line (109525) that was used as the transformation stock was homozygous for the PUFA synthase system containing transgenes PFA1, PFA2, PFA3, and HetI [14].

**Fig 3:**
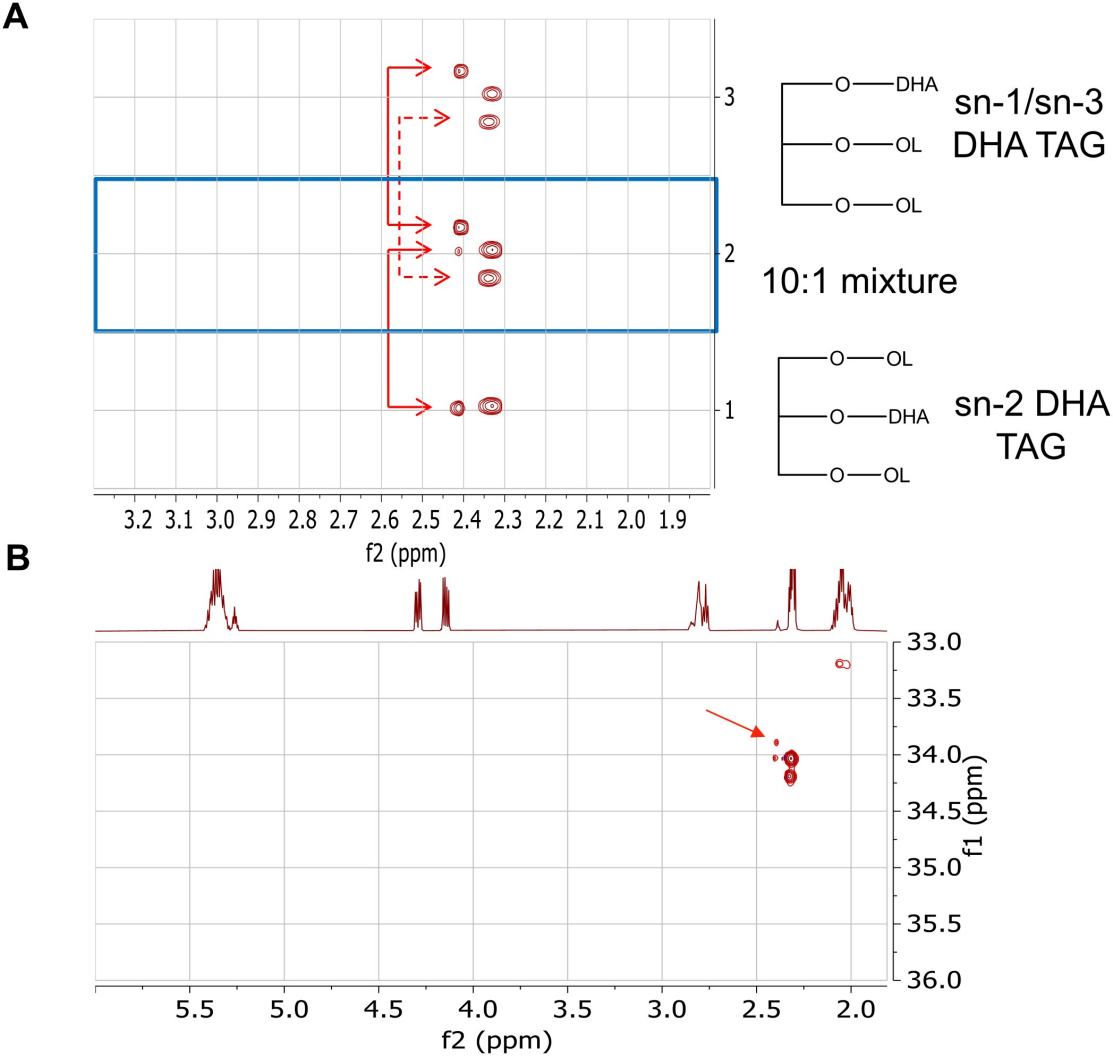
Band-selective HSQC NMR spectra. (A) Comparison of expanded HSQC spectra of pure standards, *sn*-1/*sn*-3 DHA TAG and sn-2 DHA TAG. Scale for ^13^C range: 33.6–34.5 ppm (B) Example of HSQC spectra from an Arabidopsis oil sample. The DHA gives rise to the small peaks (marked with an arrow) seen just down field (to the left) of signals from other fatty acids in the sample. The upper peak is that of the methylene group attached to *sn*-1/*sn*-3 on glycerol, whereas the lower peak arises from the methylene attached to *sn*-2 of glycerol. Relative peak volumes give the relative amounts of each.

To quantitate the amount of DHA in the *sn*-2 position on the glycerol backbone, a more sensitive NMR method was required than that used in the earlier studies. By combining ^1^H and ^13^C NMR spectra, band-selective heteronuclear single quantum coherence (HSQC) NMR was used to determine the extent of DHA incorporation into the *sn-*2 position of the glycerolipids in transgenic seed oil extracts. Custom-made TAG standards with DHA at the *sn-*1/*sn-*3 or *sn-*2 positions (and a 10:1 *sn1*/*sn-3*:*sn-*2 mixture) were used to validate this method (Fig 3A). The TAG fraction was isolated from 50 mg Arabidopsis T_2_ seed from selected single copy events. An example of the 2-D NMR spectra from a sample is shown in Fig 3B. The two peaks indicate the presence of DHA at the *sn-*2 and *sn-*1/*sn-*3 positions. These peak volumes were calculated to indicate the relative percentage of each, and projections through these peaks were also collected for comparison between samples. The larger peaks were from the other TAGs in the sample, also separated by the glycerol substitution position.

There was a significant enhancement of DHA at the *sn-*2 position compared to the *sn*-1 or *sn*-3 position of TAG in the bulk T_2_ seed oil from events expressing SzLPAAT2 FL, SzLPAAT3 FL, SzLPAAT4, Gm02LPAAT, and Gm10LPAAT, compared to the 109525 control seed with no LPAAT transgene (Table 1). The T_2_ seed from SzLPAAT3 FL events had an average of 51% of the total DHA at the *sn-*2 position (Table 1). Detection via LC-MS/MS of protein-specific peptides in these selected events confirmed the presence of SzLPAAT1 and SzLPAAT3 (FL and tr versions), SzLPAAT4, Gm02LPAAT, and Gm10LPAAT proteins in T_2_ seed from their associated constructs and the SzLPAAT2 protein in T_3_ homozygous seed.

**Table 1:** Positional analysis of DHA in T_2_ transgenic LPAAT seed TAG.

Transgene	Mean *sn-*2 DHA (%)	Standard Error	Number of Events	*p*-Value
109525 Control	23	2.8	6	1.0000
SzLPAAT1 FL	23	4.8	2	1.0000
SzLPAAT1 tr	22	4.0	3	1.0000
SzLPAAT2 FL	40*	4.0	3	0.0114
SzLPAAT2 tr	30	4.0	3	0.6892
SzLPAAT3 FL	51*	4.0	3	< .0001
SzLPAAT3 tr	21	4.0	3	1.0000
SzLPAAT4	47*	3.4	4	< .0001
Gm02LPAAT	36*	3.4	4	0.0380
Gm10LPAAT	37*	3.4	4	0.0224

The TAG fraction was isolated from T_2_ bulk seed oil and the positional analysis of DHA was determined by band-selective HSQC NMR. Percentages are relative to the total amount of DHA. Asterisks (*) indicate significantly different from the 109525 Control sample, mean comparison using Dunnett’s Method, *p* < 0.05.

The DHA canola oil tested using the qualitative ^1^H NMR method was retested using the quantitative HSQC NMR method. From the ^1^H NMR spectrum, we calculated there to be 3.7% total DHA (S2 Fig), similar to 3.5% obtained by GC-FID quantitation [14]. Integration from the 2-D spectra showed that less than 3% of the total DHA is at the *sn-*2 position (S2 Fig; [14]). Thus, both NMR methods indicate that canola contains minimal DHA at the *sn-*2 position and that the canola acyltransferases responsible for *sn-*2 acylation of TAG are selective against DHA-CoA substrates.

**Fig 4:**
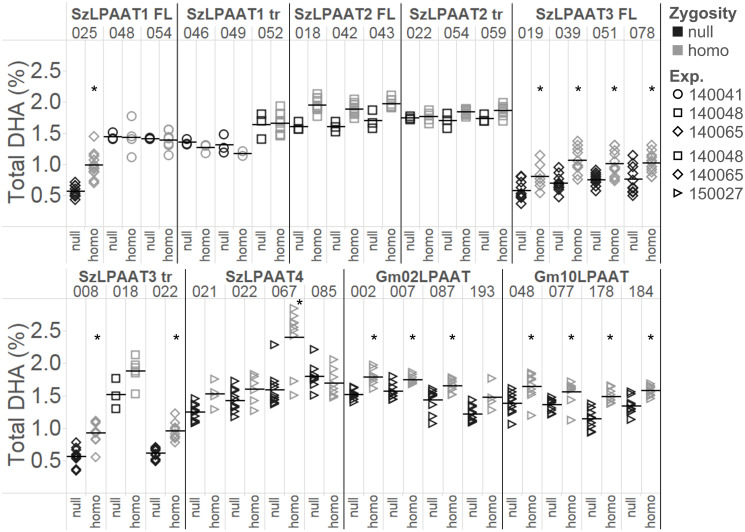
DHA (C22:6) levels of T_3_ LPAAT homozygous seed determined by GC-FID. Seed from single insertion events in the PUFA synthase background were compared with segregating null seed. Percentages are in weight % of total fatty acid methyl esters. The horizontal line represents the mean for each event. See S1 File for full fatty acid composition. Asterisks indicate there was a significant increase in the homozygous lines (gray) compared to the null lines (black) per event using the Student’s *t*-test (*p* < 0.05). Statistics were not conducted for events with less than 5 homozygous or null lines. Shapes differentiate the planting batch experiment.

Minimal differences in total DHA content were observed in bulk Arabidopsis T_2_ seed (S3 Fig) despite the increases in DHA at the *sn*-2 position of TAG, most likely due to the presence of segregating null seeds. To explore these phenotypes further, three or four independent single-copy events from each construct were selected and grown to the next generation. The plants were genotyped for transgene zygosity, and the T_3_ seed was analyzed by GC-FID for total DHA content (see S1 File for the complete fatty acid profile). The total DHA content in the homozygous T_3_ seed from certain events of SzLPAAT1 FL, SzLPAAT3 FL, SzLPAAT3 tr, SzLPAAT4, Gm02LPAAT, and Gm10LPAAT were significantly increased compared to the segregated null seed (Fig 4). All the SzLPAAT3 FL and Gm10LPAAT events had total DHA levels that were significantly increased in the homozygous lines compared to the null lines. Not all events of SzLPAAT4 and SzLPAAT3 tr showed a significant increase in DHA. SzLPAAT4 event #067 had the highest DHA content of all the homozygous lines, with almost 3% DHA. Events analyzed from planting batch experiments 140041 and 140048 (including both version of SzLPAAT2, SzLPAAT1 tr, and several SzLPAAT3 tr and SzLPAAT1 FL events) only had three null plants saved (Fig 4), so these and other events with less than five homozygous or null lines were not included in statistical analyses. The average DHA content in the segregating null (109525 background) varied, most likely due to the growth and environmental conditions from different experiments on different dates. Lipid profile and fatty acid amounts can be environmentally dependent, especially due to changes in light and temperature [40, 41].

**Fig 5:**
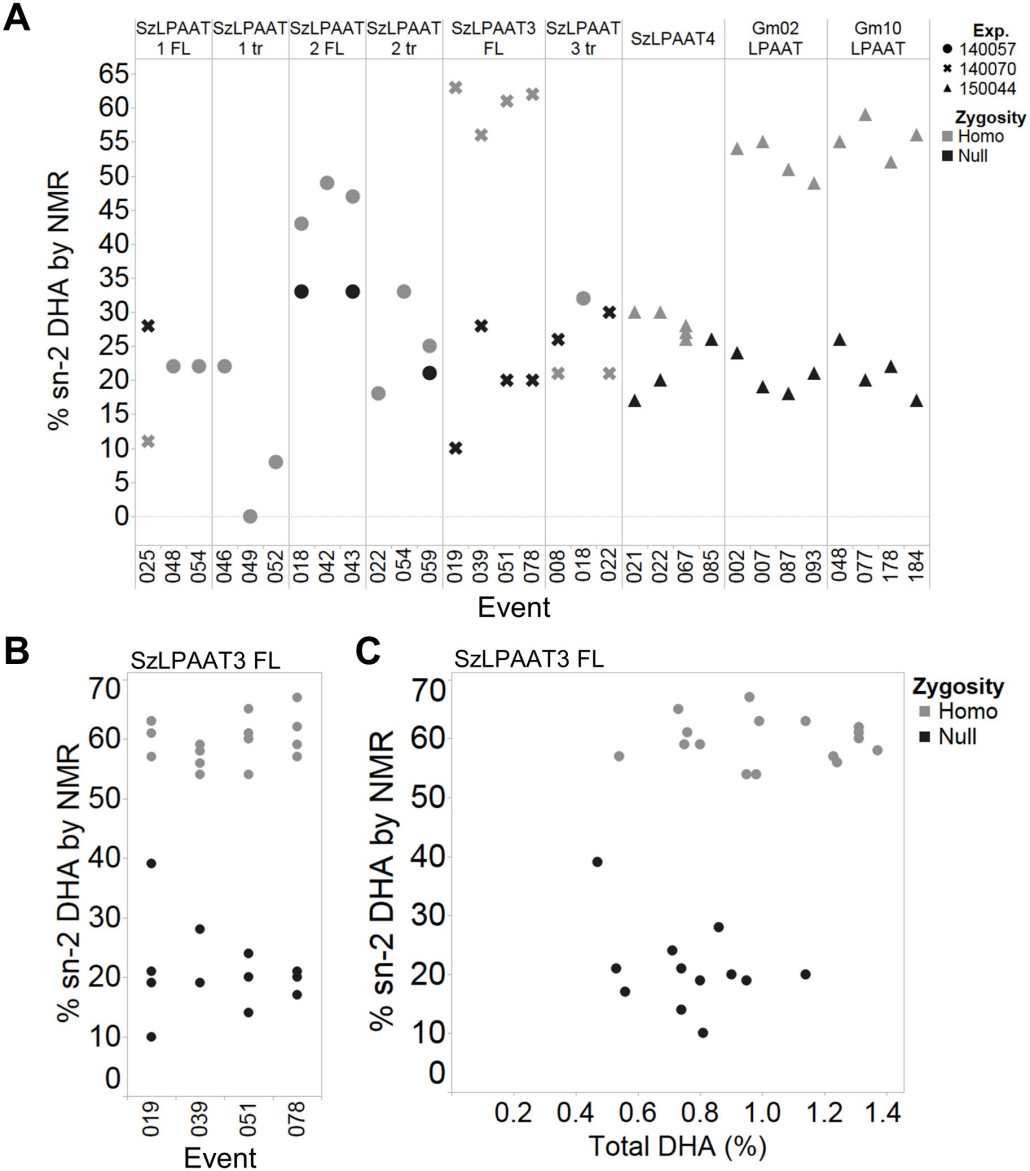
Positional analysis of DHA in T_3_ seed oil determined by band-selective HSQC NMR. **(A)** Positional analysis of DHA from T_3_ homozygous and null lines. Three to four independent events from each construct were analyzed. Shapes differentiate the planting batch experiment. **(B)** Positional analysis of DHA in additional T_3_ SzLPAAT3 FL lines. Two high DHA-producing lines, a medium DHA-producing line, and a low DHA-producing line were selected from the homozygous and null lines for positional analysis. **(C)** Comparison of total DHA determined by GC-FID and relative percent *sn*-2 DHA TAG determined by band-selective HSQC NMR for T_3_ SzLPAAT3 FL lines.

The top DHA-producing T_3_ lines from each event were then subjected to band-selective HSQC NMR to determine the extent of DHA incorporation into the *sn-*2 position of the glycerolipids. Homozygous Gm10LPAAT seed had 56% of the total DHA at the *sn-*2 position, a 148% increase over the null seed; homozygous Gm02LPAAT seed had 52% *sn-*2 DHA corresponding to a 133% increase over the nulls; SzLPAAT2 FL seed had 46% *sn-*2 (107% increase over the nulls); and SzLPAAT4 had 29% *sn-*2 DHA (30% increase in *sn-*2 DHA over nulls) (Fig 5A). Additional homozygous and null lines from the SzLPAAT3 FL events were selected for positional analysis, including high, medium, and low DHA-producing lines. The average *sn-*2 DHA incorporation for the homozygous lines across all SzLPAAT3 FL events was 60% of the DHA at the *sn-*2 position, while the average for the null lines for all events was 22% *sn-*2 DHA, corresponding to a 167% increase (Fig 5B). Four null lines did not have detectable levels of DHA at the *sn-*2 position and were assumed to have less than 10% *sn-*2 DHA and thus not included in Fig 5B. Interestingly, there was no correlation between total DHA levels (quantitated by GC-FID) and the proportion of *sn-*2 DHA (Fig 5C). These results demonstrate that heterologously expressed plant and algal LPAATs can preferentially acylate DHA into TAG at the *sn*-2 position *in vivo*.

### Gm02LPAAT increases *sn-*2 DHA by altering the distribution of DHA in TAG

**Fig 6:**
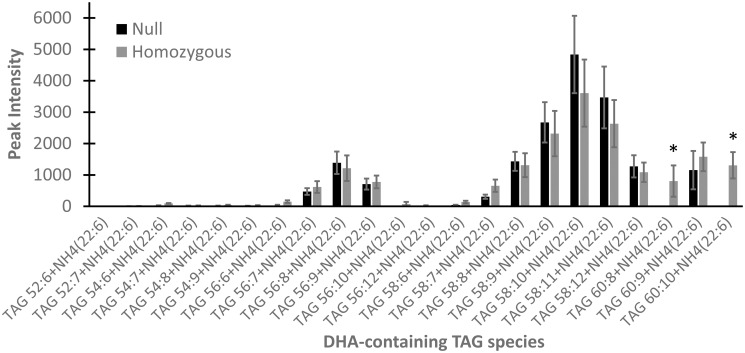
DHA-containing TAG species from Gm02LPAAT events determined by MS/MS^ALL^. Asterisks indicate novel DHA-containing TAG species detected in homozygous Gm02LPAAT events. Error bars indicate standard error of three events.

With the substantial increases in DHA at the *sn*-2 position of glycerolipids, we suspected that these LPAATs may have modified the TAG composition. Therefore, we determined the composition of DHA-containing TAG species in the seed oil from three Gm02LPAAT events using an infusion MS/MS^ALL^ workflow. This analysis indicated that there were two new species of TAGs present in the oil from Gm02LPAAT events that were not observed in the oil of corresponding event nulls (Fig 6). The two novel DHA-containing TAG species identified were TAG 60:8+NH4 and TAG 60:10+NH4. By searching this subset of DHA-TAG species with additional acyl-fragment-ion masses, we predict the three acyl chains to be 20:1, 22:6, & 18:1 for TAG 60:8+NH4 (976.8 m/z) and 20:1, 22:6, & 18:3 for TAG 60:10+NH4 (972.8 m/z). These results show that heterologous expression of a soybean LPAAT in Arabidopsis alters the TAG composition in seed oil and provides further evidence that these LPAATs have *in planta* selectivity for DHA-CoA substrate.

## Discussion

In canola, the *sn-*2 position of TAG has very low amounts of long-chain fatty acids and LC-PUFAs [22, 24, 42, 43]. To increase total DHA accumulation in plants expressing the PUFA synthase system [14], we focused on increasing DHA assembly into the *sn-*2 position of TAG by co-expressing LPAAT enzymes with the PUFA synthase system. By characterizing acyltransferases *in vitro* and *in vivo* from *Schizochytrium* and soybean, we identified novel LPAAT enzymes from both species capable of acylating the *sn*-2 position of TAG with up to two-thirds of the total amount of DHA present in seed oil.

*In vitro* measurement of LPAAT enzyme activity is challenging; eukaryote LPAATs are bound to the ER membrane or to the thylakoid membrane of the plastid [44]. Typically, measurements of LPAAT activity are performed with a heterogeneous microsomal preparation, containing either heterologously expressed protein [26, 45–47] or natively expressed protein [21, 27, 46, 48]. To mitigate background acyltransferase activity, the *slc1Δ* or *ale1Δ* (also referred to as *slc4Δ*) yeast mutants lacking LPAAT have been used to heterologously express plant acyltransferase proteins [49]. However, these mutants do not completely lack LPAAT activity due to overlapping functions and the double *slc1Δ slc4Δ* mutant is lethal [45, 50]. Specific activity of flax (*Linum usitatissimum*) LPAATs toward different acyl-CoAs was measured from membrane protein extracts by complementing the *E*. *coli* LPAAT mutant JC201 [51]. Alternatively, a cell-free wheat-germ system was used to express active *C*. *reinhardtii* LPAAT2 protein, which has unique specificity [52]. In this study, we used a Baculovirus-insect cell system to heterologously express the SzLPAATs and GmLPAAT proteins. While precise kinetic parameters were not determined, the SzLPAAT3, SzLPAAT1, and Gm02LPAAT proteins were all shown to have LPAAT activity and not LPCAT activity (Fig 2B). The *B*. *napus* DGAT1 retains some activity through solubilization in DDM detergent and affinity purification, although activity was greatly decreased [53]. We assume the DDM detergent caused a similar reduction in activity for the LPAATs, especially for SzLPAAT1 where activity was barely detected (Fig 2B). To our knowledge, this study is the first example of affinity purification of active plant and algal LPAAT proteins.

The SzLPAAT3 protein was able to use several acyl-CoA substrates, including palmitoleoyl-CoA, stearoyl-CoA, oleoyl-CoA, and DHA-CoA at similar rates under our assay conditions (Fig 2C). Plastidial LPAAT from *Brassica* species are more active on 16:0-CoA than 18:1-CoA *in vitro* [44], whereas ER membrane LPAATs have a higher substrate preference for 18:1-CoA compared to 16:0-CoA [46]. *Brassicaceae* species LPAATs can use DHA-CoA as a substrate, however *B*. *napus* LPAAT and Arabidopsis LPAAT have relatively low selectivity for DHA-CoA substrate in competition-based assays [26], which is consistent with the little or no DHA at the *sn-*2 position in transgenic *Brassica* species [14, 25]. The preference of Arabidopsis LPAAT for DHA-CoA is less than that of the *B*. *napus* LPAAT, yet the Arabidopsis LPAAT has greater selectivity for DHA-CoA in competition-based assays [26], corroborating the larger proportion of *sn*-2 DHA observed in Arabidopsis compared to canola in our study (22% versus <3%; Fig 5 and S2 Fig). Shrestha *et al*. [26] found that fungal LPAATs have a higher preference for DHA-CoA substrates than the *Brassicaceae* LPAATs. It will be of interest to compare the *M*. *alpina* LPAAT with SzLPAATs and GmLPAATs. A recently reported and very sensitive LC-MS/MS enzyme assay can allow for rapid non-radioactive competitive assays with multiple substrates to determine the selectivity of LPAATs *in vitro* [54] and could be used for these types of studies. Another reported LPAAT with high specificity for DHA-CoA is the mouse LPAAT4, which is presumed to be responsible for maintaining DHA levels in neural tissues [55]. Our *in vitro* results indicate that SzLPAAT3 has DHA-CoA activity and may be useful in increasing DHA incorporation at the *sn-*2 position of glycerolipids in oilseed crops.

Determining the regiochemistry of fatty acids esterified to TAG and the relative amount of a given fatty acid at each position is challenging and labor-intensive. The standard method involves enzymatic digestion of TAG using specific lipases to digest the acyl-chains with subsequent analysis of fatty acid methyl esters via GC-FID. This method was used to analyze DHA in transgenic *Camelina* [10] and *Brassica juncea* [25]. Initially, we used ^1^H NMR to detect *sn-*2 DHA in TAG of several species expressing the PUFA synthase system, including canola, soybean, Arabidopsis, and yeast as well as the native organism *Schizochytrium* that synthesizes DHA via the PUFA synthase system (S1B Fig). This qualitative method provided a preliminary screen to identify which transgenic organisms were acylating the *sn-*2 position of glycerolipids with DHA but was not sensitive enough to screen or determine relative proportion of DHA at the *sn-*2 position of glycerolipids in LPAAT and PUFA synthase co-expression Arabidopsis lines. By combining the advantages of ^13^C and ^1^H NMR spectra to gain more resolution and sensitivity, a band-selective HSQC NMR method for detecting *sn-*2 DHA was developed (Fig 3) [56–59]. Two-dimensional NMR has been used to examine the structure of glycerolipids [60] and assign olefinic resonances in omega-3 PUFAs [61]. In transgenic canola expressing PUFA synthase, less than 3% of the total DHA was at the *sn-*2 position (S2 Fig; [14]), consistent with other observations in *Brassicaceae* crops engineered to produce LC-PUFAs, such as *Camelina* [10, 11], *B*. *juncea* [25], and Arabidopsis [9]. Our 2-D NMR method provides a much more robust and sensitive analysis for determining the relative amount of DHA at the *sn-*2 position than ^13^C or ^1^H NMR alone and is much less labor intensive than previously used lipase methods. Further improvements in sensitivity can be made by using a small volume micro-cryoprobe to decrease the amount of sample required and the acquisition time.

The soybean LPAATs Gm02LPAAT and Gm10LPAAT significantly increase total DHA levels in T_3_ Arabidopsis seed when co-expressed with the PUFA synthase system, compared to sibling nulls for each event (Fig 4). These results, and an average of 22% *sn*-2 DHA present in the Arabidopsis nulls (Fig 5), demonstrate that the native plant LPAATs can use DHA-CoA as a substrate to acylate the *sn*-2 position of TAG. The accumulation of 60% *sn*-2 DHA in the homozygous SzLPAAT3 FL lines (Fig 5A) suggests SzLPAAT3 is predominantly acylating the *sn-*2 position of the glycerolipids with DHA-CoA. The high accumulation at this position may in part be due to high level of SzLPAAT3 expression driven by the strong seed-specific phaseolin promoter compared to the expression of endogenous Arabidopsis acyltransferases. Heterologous expression of a fungal *M*. *alpina* LPAAT in DHA-producing transgenic Arabidopsis also increased the proportion of *sn-*2 DHA up to 48% of total DHA [26]. Even though Arabidopsis can acylate the *sn-*2 position of glycerolipids with DHA-CoA, the native Arabidopsis acyltransferases appear to be selective against *sn-*2 DHA acylation because *sn-*2 DHA is less than one third of total DHA, as expected for random non-selective placement at any one of the three positions of the glycerolipid.

Interestingly, expression of the soybean LPAAT altered the distribution of DHA found in TAGs, as two new DHA-containing TAG species (Fig 6) were identified in these seed oils. The transgenic LPAATs could be outcompeting endogenous acyltransferases for *de novo* DHA-CoA or they could be using the DHA-CoA made available from the PC pool (via acyl editing) to re-esterify DHA to TAG. No di-DHA TAG or tri-DHA TAG species were detected (Fig 6), although these were found with the fungal *M*. *alpina* LPAAT co-expressed with the desaturase-elongase LC-PUFA pathway [26]. Transgenic *Camelina* expressing a desaturase-elongase LC-PUFA pathway accumulate detectable amounts of tri-DHA TAG (66:18) [62] without an exogenous LPAAT, indicating that *Camelina* acyltransferases (like Arabidopsis) can acylate the *sn*-2 position of TAG. The two new DHA-containing TAG species identified in Gm02LPAAT seed oil (Fig 6) provide further evidence that these LPAATs have selectivity for DHA-CoA substrates *in planta* and have different specificity than the host Arabidopsis LPAATs.

Although these plant and algal LPAATs increase the total amount of DHA and *sn*-2 DHA in Arabidopsis using the PUFA synthase system, the levels of total DHA are still somewhat less than those attained using the desaturase-elongase LC-PUFA pathway expressed in Arabidopsis, which can produce up to 21% DHA in seed oil [9]. This difference indicates that there is potential to further improve DHA synthesis or availability of DHA-CoA from the PUFA synthase system expressed in plants. Nevertheless, we anticipate the plant and algal LPAATs that we have characterized could also increase total and *sn*-2 DHA in seed oils that produce DHA via the desaturase-elongase LC-PUFA pathway.

The importance of finding sustainable sources of DHA is due to increasing demand driven by the many health benefits of high omega-3 LC-PUFA diets [1, 2, 63, 64]. A benefit of using the PUFA synthase system to produce DHA in transgenic oilseeds is that it synthesizes DHA directly from malonyl-CoA in the cytosol. This complementation of the fatty acid profile allows the PUFA synthase DHA trait to be stacked with other heart-healthy oilseed traits such as high oleic acid oils [14]. Engineering preferential placement of DHA at the *sn-*2 position of glycerolipids in canola will increase the low amounts of DHA currently present at this position (S2 Fig; [10, 14, 25]). Soybean, especially new high oleic acid varieties, may be a suitable oilseed crop for producing DHA, as our results show that native soybean LPAATs can use DHA-CoA relatively well as a substrate. Over-expression of LPAATs such as Gm02LPAAT in transgenic DHA-accumulating soybeans may further increase total and *sn*-2 DHA. Although the relative bioavailability of specific fatty acids at the *sn*-2 position of dietary TAG is unclear, some studies indicate that *sn*-2 DHA is readily taken up in mammalian tissues [65, 66]. Increasing the amount of DHA in the *sn-*2 position could therefore improve dietary availability of plant-produced DHA, as well as the total DHA content of oilseeds.

## Supporting information

S1 Fig Proton NMR analysis. **(A)** Proton NMR spectrum of DHA TAG standards. **(B)** Qualitative ^1^H NMR of oils from custom standards, native *Schizochytrium* organism, and from several host systems accumulating DHA via expression of the four PUFA synthase transgenes. The red line indicates the position of the DHA methylenes (C-2 and C-3). The asterisks indicate samples containing a mixture of *sn*-1/*sn*-3 and *sn*-2 substituted DHA. Scale is 2.25 to 2.45 ppm.
(TIF)

S2 Fig NMR of canola oil. Two-dimensional (2-D) band-selective HSQC NMR of bulk canola oil extract containing DHA. Scale for ^1^H range is 0.0 to 12.5 ppm (left) and for the 2-D spectrum (right) it is 2.15 to 2.80 ppm and the ^13^C scale is 33.80 to 34.30 ppm.
(TIF)

S3 Fig DHA (C22:6) levels (in weight %) of bulk T_2_ segregating seed. The LPAATs were expressed in the PUFA Synthase background 109525.
(TIF)

S1 Table Primers and probes used for quantitative PCR to determine copy number of transgenes. (PDF)

S2 Table MS/MS^ALL^ parameters. Chromatographic and mass spectral parameters for lipid analysis by direct infusion using a 5600 Q-TOF (Sciex) equipped with a Nexera X2 UHPLC (Shimadzu).
(PDF)

S1 File Quantitation of complete fatty acid profile of T3 seed, measured by GC-FID. Values are in weight % of total fatty acid methyl esters.
(XLSX)
