# Supplementary figures and images for: Plant and algal lysophosphatidic acid acyltransferases increase docosahexaenoic acid accumulation at the sn-2 position of triacylglycerol in transgenic Arabidopsis seed oil
Source: PLoS One. 2021 Aug 25;16(8):e0256625. doi: 10.1371/journal.pone.0256625 (PMC8386867; doi:10.1371/journal.pone.0256625)

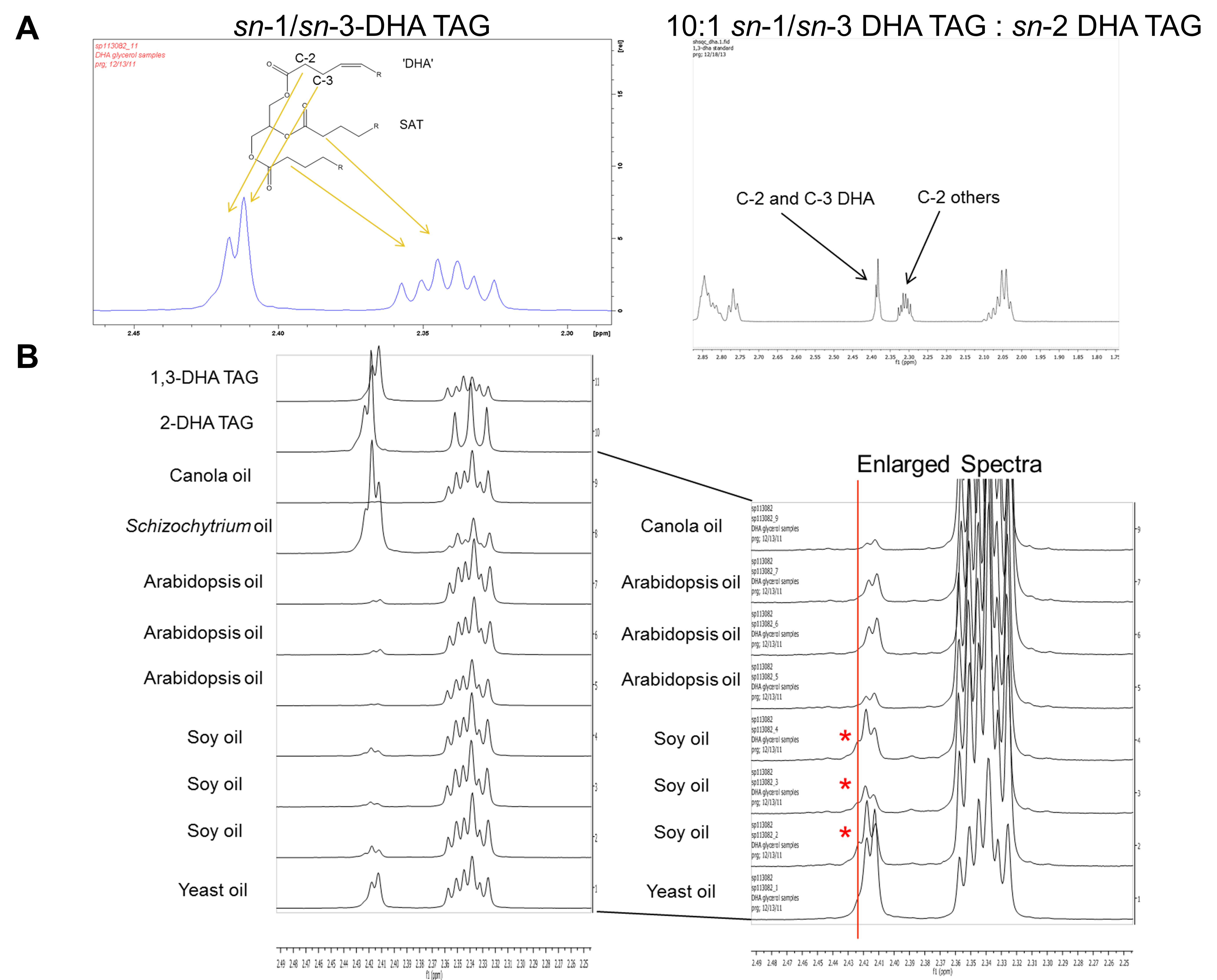

Supplement: S1 Fig — (A) Proton NMR spectrum of DHA TAG standards. (B) Qualitative 1H NMR of oils from custom standards, native Schizochytrium organism, and from several host systems accumulating DHA via expression of the four PUFA synthase transgenes. The red line indicates the position of the DHA methylenes (C-2 and C-3). The asterisks indicate samples containing a mixture of sn-1/sn-3 and sn-2 substituted DHA. Scale is 2.25 to 2.45 ppm. (TIF) [file pone.0256625.s001.tif]

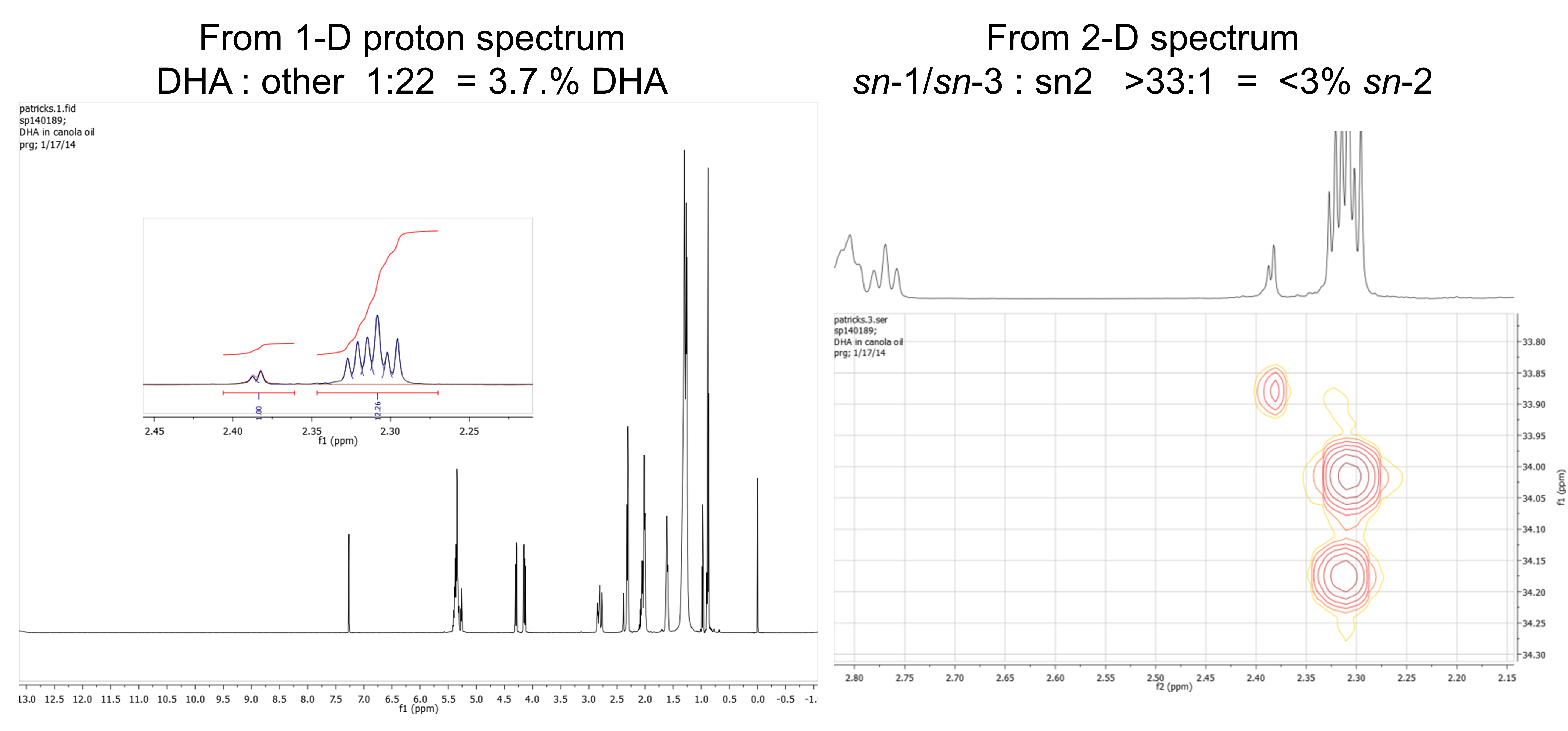

Supplement: S2 Fig — Two-dimensional (2-D) band-selective HSQC NMR of bulk canola oil extract containing DHA. Scale for 1H range is 0.0 to 12.5 ppm (left) and for the 2-D spectrum (right) it is 2.15 to 2.80 ppm and the 13C scale is 33.80 to 34.30 ppm. (TIF) [file pone.0256625.s002.tif]

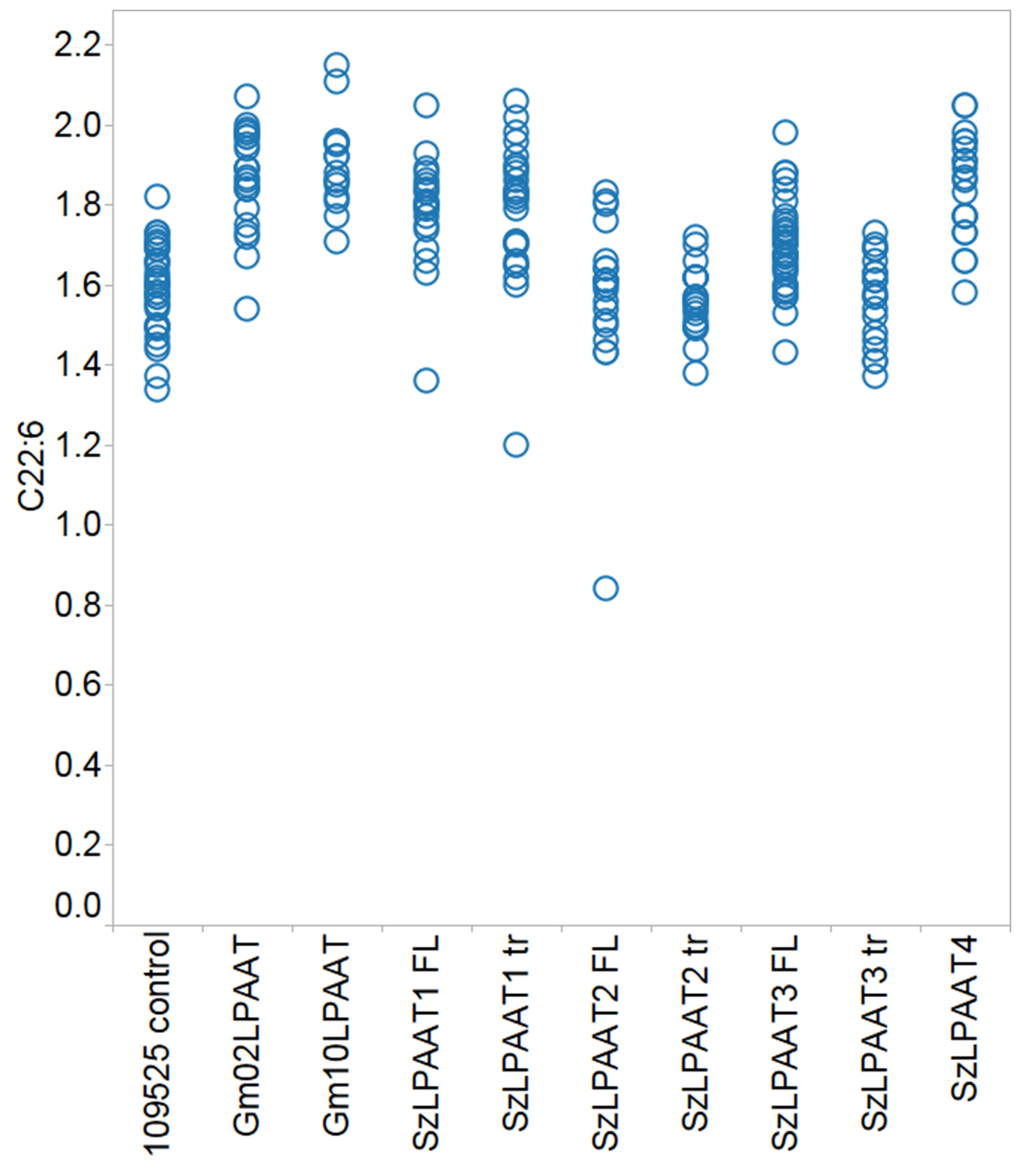

Supplement: S3 Fig — The LPAATs were expressed in the PUFA Synthase background 109525. (TIF) [file pone.0256625.s003.tif]
