# Supplementary material for: Plant and algal lysophosphatidic acid acyltransferases increase docosahexaenoic acid accumulation at the sn-2 position of triacylglycerol in transgenic Arabidopsis seed oil
Source: PLoS One. 2021 Aug 25;16(8):e0256625. doi: 10.1371/journal.pone.0256625 (PMC8386867; doi:10.1371/journal.pone.0256625)
Supplement: S1 Table — (PDF) [file pone.0256625.s004.pdf]

S1 Table. Primers and probes used for quantitative PCR to determine copy number of transgenes.

Target	Left Primer	Left Primer Sequence	Right Primer	Right Primer Sequence	Probe	Probe Sequence
AAD12	AAD12v1 F	CAGAGTCCATGCTCACCAAT	AAD12v1 R	CACGTGGCAACTTGAAATCC	Cy5	TGGAGATGTGGTTGTGTGGGACAA
SzLPAAT1	poSzLPAAT1 F	GAGTGGCTGTTTCTCGTCTT	poSzLPAAT1 R	CTTGGAGGTCTCTTGGTTGATAG	FAM	ACACGGCTGAAGATTGCCTCAGAA
SzLPAAT2	poSzLPAAT2 F	CGGTTGAACTTGTCAGACCTAT	poSzLPAAT2 R	CTCCACTGTTGGGAAGATTGA	FAM	AGGCGTTCATGAAGGGTCCAAGAA
SzLPAAT3	poSzLPAAT3 F	CAGCAAGGTATCTGAGCAAGAG	poSzLPAAT3 R	ACGTAAGGTCAAACCTGCAATA	FAM	AAGATGCCAGGAACATCAGGGAAGG
TAFII15	TAFII15-F	GAGGATTAGGGTTTCAACGGAG	TAFII15-R	GAGAATTGAGCTGAGACGAGG	HEX	AGAGAAGTTTCGACGGATTTCGGGC
SzLPAAT4	SzLPAAT4 v2 F	CGGATCGAGAGCTTGAAAGAG	SzLPAAT4 v2 R	CGATGGCCTTGGATTCATAGAT	FAM	AACATCAACAGCCTCCTCTGTGGT
GmLPAAT	GmLPAAT F	CCTTCCAGGAGGATAGTAGTGA	GmLPAAT R	GACGACCCGTAAAGTCTCTTG	FAM	CGTAACAACGACACAAGCCCACAA
