# Supplementary material for: Plant and algal lysophosphatidic acid acyltransferases increase docosahexaenoic acid accumulation at the sn-2 position of triacylglycerol in transgenic Arabidopsis seed oil
Source: PLoS One. 2021 Aug 25;16(8):e0256625. doi: 10.1371/journal.pone.0256625 (PMC8386867; doi:10.1371/journal.pone.0256625)
Supplement: S2 Table — Chromatographic and mass spectral parameters for lipid analysis by direct infusion using a 5600 Q-TOF (Sciex) equipped with a Nexera X2 UHPLC (Shimadzu). (PDF) [file pone.0256625.s005.pdf]

S2 Table. MS/MS^{ALL} parameters. Chromatographic and mass spectral parameters for lipid analysis by direct infusion using a 5600 Q-TOF (Sciex) equipped with a Nexera X2 UHPLC (Shimadzu).

Parameter	Setting
Column	loop injection infusion
Mobile Phase	Methanol:dichloromethane (1:1 v/v) + 5 mM ammonium acetate
Flow rate gradient	6-30 µL/min
Wash	Methanol:dichloromethane (1:1 v/v) + 5 mM ammonium acetate
Ion source gas 1 & 2	20 & 25
Curtain Gas™	20
Temperature	150°C
Ion spray voltage	4200-5200
Declustering Potential	60
Scan event 1	TOF MS
Scan event 2	MS/MS^{ALL}
